# Outcomes of patients with rodenticide poisoning at a far east poison center

**DOI:** 10.1186/2193-1801-2-505

**Published:** 2013-10-03

**Authors:** Hsin-Ying Yu, Ja-Liang Lin, Jen-Fen Fu, Jui-Hsiang Lin, Shou-Hsuan Liu, Cheng-Hao Weng, Wen-Hung Huang, Kuan-Hsing Chen, Ching-Wei Hsu, Tzung-Hai Yen

**Affiliations:** Department of Nephrology and Division of Clinical Toxicology, Chang Gung Memorial Hospital, 199 Tung Hwa North Road, Taipei, 105 Taiwan; Department of Medical Research, Chang Gung Memorial Hospital, Linkou, Taiwan; College of Medicine, Chang Gung University, Taoyuan, Taiwan; Department of Nephrology, Taoyuan General Hospital, Taoyuan, Taiwan

**Keywords:** Brodifacoum, Bromadiolone, Rodenticide, Superwarfarin, Poisoning, Vitamin K1

## Abstract

**Background:**

Rodenticide poisoning remains a major public health problem in Asian countries. Nevertheless, very few data are available in world literature regarding the outcomes of these patients. Therefore, the purpose of this study was to investigate the clinical outcomes of rodenticide poisonings in our hospital and to compare these data with published reports from other international poison centers.

**Findings:**

We retrospectively examined the records of 20 patients with rodenticide poisoning (8 brodifacoum, 12 bromadiolone) who were referred to Chang Gung Memorial Hospital between 2000 and 2011. It was found that most of the rodenticide patients were middle-aged adults. Both genders were equally affected and many patients had a past history of major depressive disorder or schizophrenia. Nevertheless, patients with bromadiolone were referred significantly sooner than patients with brodifacoum poisoning (0.1 ± 0.1 versus 5.5 ± 10.5, P < 0.001). Furthermore, it was found that patients with brodifacoum suffered higher incidences of ecchymosis (50.0% versus 0%, P = 0.006) and hematuria (50.0% versus 0%, P = 0.006) than patients with bromadiolone poisoning. Laboratory analysis also demonstrated a poorer hemostatic profile of patients with brodifacoum [prothrombin time (PT), international normalized ratio (INR), 4.3 ± 4.8 versus 1.0 ± 0.1, P = 0.032; PT prolongation, 50.0% versus 0%, P = 0.006; activated partial thromboplastin time (aPTT) prolongation, 50.0% versus 0%, P = 0.006] than patients with bromadiolone poisoning. At the end of analysis, no patient died of the poisoning.

**Conclusion:**

The favorable outcome (zero mortality rate) is comparable to the published reports from other international poison centers. Further studies are warranted.

## Introduction

Superwarfarin rodenticide consists of two classes of compounds, the 4-hydroxycoumarins and the indandiones (Gunja et al. 2011). These compounds have replaced warfarin as an anticoagulant rodenticide. Given that the 4-hydroxycoumarin derivatives brodifacoum and bromadiolone are now the most frequently used rodenticides in Taiwan, they also account for the majority of rodenticide poisonings reported to our poison center.

Both brodifacoum and bromadiolone act by the same mechanism as warfarin but are 100 times more potent and have much longer serum and tissue half-lives (Park and Leck 1982). Coagulation factors II, VII, IX, X, and other vitamin K–dependent proteins require gamma-carboxylation of glutamic acid residues to be biologically active. In the carboxylation reaction, the cofactor vitamin K is converted to an inactive vitamin K-2,3-epoxide. Active vitamin K is regenerated by epoxide reductase enzymes, which are blocked by warfarin and its analogs (Furie et al. 1999). The limited availability of active vitamin K produces a bleeding diathesis or complication.

The increased efficacy of superwarfarins as rodenticides results from their high lipid solubility, affinity for hepatic tissue and slow elimination from the body. The treatment of poisoned patients includes gastric lavage, followed by infusion of active charcoal, vitamin K1 therapy, and FFP transfusion in the case of bleeding (Caravati et al. 2007).

Rodenticide poisoning remains a major public health problem in Asian countries. Nevertheless, very few data are available in literature regarding the outcomes of these patients. Therefore, the purpose of this study was to investigate the clinical outcomes of these poisonings in our hospital and to compare these data with published reports from other international poison centers.

## Materials and methods

### Ethics

This retrospective observational study complied with the guidelines of the Declaration of Helsinki and was approved by the Medical Ethics Committee of Chang Gung Memorial Hospital, a tertiary referral center located in the northern part of Taiwan. All primary data were collected according to the Strengthening the Reporting of Observational Studies in Epidemiology guidelines.

### Patients

We examined the records of 20 patients with rodenticide poisoning who were examined at Chang Gung Memorial Hospital between 2000 and 2011. Demographic, clinical and laboratory data were collected, and the mortality rate was analyzed. Diagnosis of rodenticide intoxication was based on exposure history, and confirmed by clinical manifestations as well as physical and laboratory examination.

### Inclusion and exclusion criteria

All patients who were diagnosed with rodenticide poisoning at Chang Gung Memorial Hospital between 2000 and 2011 were eligible for inclusion in this study. The type of rodenticide was verified by container or packaging information. Patients were excluded if the type of toxin swallowed was unknown even if there was a clinical suspicion of rodenticide intoxication. Finally, patients with co-ingestants or physical injury were also excluded from this analysis.

### Detoxification protocol

The protocols used to treat patients included gastric lavage (in case of within 4 hours of exposure), followed by infusion of 1 g/kg activated charcoal and 250 mL magnesium citrate via nasogastric tube. Magnesium citrate was used to prevent constipation after charcoal administration. At our hospital, the treatment of rodenticide poisoning was to administer vitamin K1 via an intramuscular route because the oral form of vitamin K1 was unavailable. Intravenous vitamin K1 infusion was avoided if possible due to the risk of anaphylaxis with this route of administration. Acute doses of prophylactic vitamin K were also given at emergency department to patients with a known ingestion of rodenticide who was asymptomatic, but the antidote was discontinued as soon as confirmation of no coagulopathy or bleeding. Finally, a transfusion of FFP was indicated for patients with active bleeding.

### Definitions of clinical events

An ecchymosis was defined as a subcutaneous purpura of larger than 1 cm. A subconjunctival hemorrhage was defined as a bleeding underneath the conjunctiva. Hypotension was defined as a systolic blood pressure of less than 90 mmHg or diastolic blood pressure of less than 60 mmHg. Melena was defined as the discharge of black, tarry, bloody stools, usually resulting from a hemorrhage in the alimentary tract. Hematuria was defined as presence of blood in the urine. Prolongation of the PT and aPTT were based on an INR value >1.5 (Stanworth et al. 2011), or aPTT value of > 1.5 times (Eby 1997) the laboratory mean control.

### Statistical analysis

Continuous variables are expressed as means and standard deviations and categorical variables as numbers with percentages in brackets. All data were tested for normality of distribution and equality of standard deviations before analysis. For comparisons between patient groups, we used Student’s *t* test for quantitative variables and Chi-square or Fisher’s exact tests for categorical variables. We considered results that rejected the null hypothesis with 95% confidence to be significant. All analyses were performed with IBM SPSS Statistics Version 20.

## Results

Most of the rodenticide patients were middle-aged adults (Table 1). Both genders were equally affected and many patients had a past history of major depressive disorder or schizophrenia. Furthermore, it was found that patients with bromadiolone were referred significantly sooner than patients with brodifacoum poisoning (0.1 ± 0.1 versus 5.5 ± 10.5 hours, P < 0.001).

**Table 1:** **Baseline characteristics of patients with brodifacoum and bromadiolone rodenticide poisoning (N = 20)**

Variable	Bromadiolone (N = 12)	Brodifacoum (N = 8)	P value
Age, year	37.3 ± 25.7	32.3 ± 17.7	0.639
Female, n (%)	6 (50.0)	5 (62.5)	0.582
Intentional, n (%)	9 (75)	7 (87.5)	0.494
Diabetes mellitus, n (%)	2 (16.7)	0 (0)	0.224
Hypertension, n (%)	3 (25.0)	0 (0)	0.125
Major depressive disorder and schizophrenia, n (%)	8 (66.7)	7 (87.5)	0.292
Alcohol consumption, n (%)	6 (50.0)	1 (12.5)	0.085
Smoking habit, n (%)	5 (41.7)	3 (37.5)	0.852
Betel nut chewing, n (%)	4 (33.3)	0 (0)	0.068
Therapeutic use of warfarin or antiplatelet drugs	0 (0)	0 (0)	1.000
Time elapsed between poisoning and hospital arrival, hour	0.1 ± 0.1	5.5 ± 10.5	<0.001***

Note: ***P < 0.001.

Reported bleeding events included ecchymosis, subconjunctival hemorrhage, melena, and hematuria (Table 2). Some patients also complained of acute gastrointestinal symptoms, for example nausea, vomiting, abdominal distension, abdominal pain, etc. After analysis, it was revealed that patients with brodifacoum suffered higher incidences of bleeding complications, such as ecchymosis (50.0% versus 0%, P = 0.006) and hematuria (50.0% versus 0%, P = 0.006), than patients with bromadiolone poisoning (Table 2). Furthermore, laboratory analysis also demonstrated poorer hemostatic profiles for patients with brodifacoum (PT, INR, 4.3 ± 4.8 versus 1.0 ± 0.1, P = 0.032; PT prolongation, 50.0% versus 0%, P = 0.006; aPTT prolongation, 50.0% versus 0%, P = 0.006) than patients with bromadiolone poisoning (Table 3).

**Table 2:** **Clinical manifestations of patients with brodifacoum and bromadiolone rodenticide poisoning (N = 20)**

Variable	Bromadiolone (N = 12)	Brodifacoum (N = 8)	P value
Body temperature, °C	36.1 ± 0.5	36.5 ± 1.1	0.331
Pulse rate, per minute	87.9 ± 21.4	98.4 ± 24.1	0.333
Respiratory rate, per minute	19.1 ± 2.1	21.0 ± 2.4	0.098
Systolic pressure, mmHg	148.4 ± 39.7	114.6 ± 24.2	0.049*
Diastolic pressure, mmHg	80.3 ± 16.8	70.4 ± 17.8	0.233
Ecchymosis, n (%)	0 (0)	4 (50.0)	0.006**
Subconjunctival hemorrhage, n (%)	0 (0)	2 (25.0)	0.068
Hypotension, n (%)	0 (0)	2 (25.0)	0.068
Nausea, n (%)	1 (8.3)	1 (12.5)	0.761
Vomiting, n (%)	2 (16.7)	2 (25.0)	0.648
Abdominal distension, n (%)	0 (0)	1 (12.5)	0.209
Abdominal pain, n (%)	4 (33.3)	2 (25)	0.690
Melena, n (%)	1 (8.3)	3 (37.5)	0.110
Hematuria, n (%)	0 (0)	4 (50.0)	0.006**

Note: *P < 0.05, **P < 0.01.

**Table 3:** **Laboratory findings of patients with brodifacoum and bromadiolone rodenticide poisoning (N = 20)**

Variable	Bromadiolone (N = 12)	Brodifacoum (N = 8)	P value
Blood test			
Red blood cell, ×10^6^//uL	4.5 ± 0.6	4.2 ± 1.3	0.422
Hemoglobin	13.1 ± 2.2, g/dL	12.0 ± 3.6	0.382
Platelet, 1000/uL	233.8 ± 62.4	257.1 ± 140.5	0.616
White blood cell, 1000/uL	6.9 ± 1.7	11.3 ± 9.7	0.143
Neutrophils, %	61.7 ± 12.7	59.6 ± 21.2	0.786
Aspartate aminotransferase, U/L	31.4 ± 17.1	28.5 ± 5.9	0.702
Alanine aminotransferase, U/L	28.6 ± 23.2	28.0 ± 29.1	0.962
Albumin, g/dL	4.0 ± 0.4	4.3 ± 0.4	0.343
Total bilirubin, mg/dL	0.7 ± 0.1	0.6 ± 0.1	0.468
Blood urea nitrogen, mg/dL	10.5 ± 4.8	13.1 ± 6.7	0.475
Creatinine, mg/dL	0.7 ± 0.3	0.8 ± 0.2	0.827
Calcium, mg/dL	9.1 ± 0.8	8.9 ± 0.6	0.730
Phosphate, mg/dL	4.0 ± 1.1	2.8 ± 0.2	0.416
Sodium, mEq/L	142.9 ± 2.5	143.2 ± 3.5	0.861
Potassium, mEq/L	4.0 ± 0.3	4.0 ± 0.3	1.000
Chloride, mEq/L	110.5 ± 7.8	108.0 ± 4.2	0.728
Glucose, mg/L	107.7 ± 34.8	110.0 ± 7.1	0.935
Cholesterol, mg/dL	135.5 ± 21.9	210.0 ± 21.0	0.220
Triglyceride, mg/dL	207.0 ± 41.0	502.0 ± 50.2	0.081
C-reactive protein mg/L	16.1 ± 1.6	7.1 ± 11.4	0.562
PT, INR	1.0 ± 0.1	4.3 ± 4.8	0.032*
PT prolongation, n (%)	0 (0)	4 (50)	0.006**
aPTT, n (%)	0 (0)	4 (50)	0.006**
Urine test			
Blood, n (%)	0 (0)	5 (62.5)	0.004**
Red blood cell, per HPF	0 ± 0	217.6 ± 264.3	0.206
Stool test			
Occult blood, n (%)	1 (8.3)	1 (12.5)	0.910

Note: Activated partial thromboplastin time, *HPF* high power field, *INR* International normalized ratio, *PT* Prothrombin time *P < 0.05, **P < 0.01.

As shown in Table 4, there were no significant differences in treatment modalities between these two groups. Finally, none of the patients died of the poisoning.

**Table 4:** **Treatment outcomes of patients with brodifacoum and bromadiolone rodenticide poisoning (N = 20)**

Variable	Bromadiolone (N = 12)	Brodifacoum (N = 8)	P
Gastric lavage, n (%)	7 (58.3)	4 (50)	0.588
Active charcoal and magnesium citrate, n (%)	6 (50.0)	2 (25.0)	0.303
Vitamin K1, n (%)	10 (83.3)	6 (75.0)	0.450
FFP, n (%)	0 (0)	3 (37.5)	0.059
Mortality, n (%)	0 (0)	0 (0)	1.000

Note: *FFP* Fresh frozen plasma.

## Discussion

There was no mortality in this study. The favorable outcome, shown in Table 4, is comparable to that of published reports from other international poison centers (Table 5), whose the mortality rate was also 0%.

**Table 5:** **Comparison of clinical outcome between current and published studies (sample size > 5) from different geographic areas**

Study	Year	Area	Sample size	Proportion of brodifacoum	Outcome	Mortality rate
Chua and Friedenberg (1998)	1998	USA	11	Brodifacoum (9/11)	Ecchymosis (1/11)	0
					Hematuria (1/11)	
Ingels et al. (2002)	2002	USA	545	Not reported	Not reported	0
Shepherd et al. (2002)	2002	USA	10762	Brodifacoum (10762/10762)	Not reported	0
Wu et al. (2009)	2009	Taiwan	9	Brodifacoum (7/9)	Ecchymosis (5/9)	0
					Hematuria (8/9)	
Hong et al. (2010)	2010	Korea	10	Brodifacoum (5/10)	Ecchymosis (4/10)	0
					Hematuria (4/10)	
Current study	2011	Taiwan	20	Brodifacoum (8/20)	Ecchymosis (4/20)	0
					Hematuria (4/20)	

Nonetheless, there are few original studies reporting outcomes of bromadione poisoning in the literature. In 1998, Chua and Friedenberg (1998) reported 11 cases of superwarfarin poisoning from northern Wisconsin, USA. All 7 children had accidentally ingested superwarfarin, 2 adults had Munchausen syndrome, and 1 teenager and 1 adult had attempted suicide using superwarfarin. Nine of the 11 cases had taken brodifacoum. None of the patients died. In another study (Ingels et al. 2002), Ingels et al. prospectively examined 595 children younger than 6 years of age who reported to the California Poison Control System with acute unintentional superwarfarin ingestions. None of the patients had clinically important coagulopathy. Two patients had an INR of 1.5 or greater (1.5 and 1.8) without symptoms. Single nosebleeds were reported in another 2 patients with normal 48-hour INRs. Another child had a small amount of blood crusted in the nose with no other symptoms and no laboratory work available. One child with a normal 48-hour INR had blood-streaked stools that were thought to be caused by an anal fissure. However, the proportion of patients taking brodifacoum was not described. In another study (Shepherd et al. 2002), the American Association of Poison Control Centers (AAPCC) data from 1993 to 1996 were retrospectively reviewed for acute, unintentional brodifacoum exposures in children aged 6 years and younger. The analysis found 10,762 cases that involved single, acute, unintentional ingestions of brodifacoum. In this cohort, there were no deaths or major effects reported. Although 67 patients reported evidence of coagulopathy, no major effects or deaths were reported. Minor and moderate effects were reported in 38 and 54 children, respectively. Approximately half of all the children received some form of gastrointestinal decontamination, which had no effect on the distribution of outcomes but which caused adverse effects in 42 patients. In another study (Wu et al. 2009), Wu et al. studied 9 Taiwanese cases of superwarfarin poisoning at the Changhua Christian Hospital, seven of which were brodifacoum intoxication. Of the 9 patients, hematuria occurred in eight. Laboratory tests showed extremely prolonged PT and aPTT, which could be corrected to normal by mixing 1:1 with normal pooled plasma; they also had very low functional levels of factor II, VII, IX, X, and proteins C and S, but normal functional levels of factors V, VIII, fibrinogen, and anti-thrombin III. Large doses of vitamin K1 were needed for 3 months or more to treat and correct the coagulopathy among the patients. The majority of the patients presented with gross hematuria, and thus required prolonged use of large doses of vitamin K1 for the treatment of superwarfarin intoxication. Finally, Hong et al. (Hong et al. 2010) found 10 Korean patients diagnosed as or highly suspicious for superwarfarin intoxication at Gachon University Gil Hospital. Bleeding symptoms varied among the patients and patients uniformly showed prolonged PT and aPTT with decreased activity of vitamin K dependent coagulation factors. Positive serum brodifacoum test results in 4 of 5 requested patients contributed to confirmatory diagnosis. High dose vitamin K1 therapy promptly corrected the prolonged PT and aPTT, but hasty discontinuation caused repeated bleeding diathesis in 6 patients. Therefore, high dose and prolonged use of vitamin K1 therapy was necessary for effective detoxification.

In this study, it was found that patients with brodifacoum suffered higher incidences of bleeding complications, such as ecchymosis (P = 0.006) and hematuria (P = 0.006), than patients with bromadiolone poisoning (Table 2). Previous laboratory studies also indicated that the acute lethal dose 50 (LD50) for these two rodenticides were quite different. For example, the acute LD50 of brodifacoum for various animals were rats 0.27-0.30 mg/kg body weight (bw), mice 0.40 mg/kg bw, rabbits 0.30 mg/kg bw, guinea-pigs 0.28 mg/kg bw, cats 25 mg/kg bw and dogs 0.25-3.6 mg/kg bw (Brodifacoum Web 2013); on the other hand, the acute LD50 of bromadiolone for various animals were rats 1.125 mg/kg bw, mice 1.75 mg/kg bw, rabbits 1 mg/kg bw, dogs >10 mg/kg bw and cats >25 mg/kg bw (Bromadiolone Web 2013). Another consideration is the speedy of hospital referral. In this study, it was found that patients with bromadiolone were referred significantly sooner than patients with brodifacoum poisoning (Table 1). Therefore, it seems reasonable to consider that patients who present very soon after oral exposure and who receive aggressive gastric decontamination procedures would be less likely to develop clinical signs and symptoms of the exposure when compared to a group that have delayed presentation to the emergency department and delays in receiving gastric emptying treatments.

There were only 20 cases of rodenticide poisonings discovered over a period of 11 years (Table 1). In contrast to numerous cases of poisonings due to other toxins (Yang et al. 2012; Liu et al. 2012; Lin et al. 2012; Lin et al. 2011), the data indicate that rodenticide exposure in Taiwan is relatively uncommon. Similarly, Berny et al. (Berny et al. 2010) also found that the number of anticoagulant exposures reported to the Lyon Poison Control Center was very limited. This might be the result of the addition of bittering agents (Mason et al. 1985) into the formulations of rodenticides, which make them less tasty. Similarly, this might help explain why most of the rodenticide cases were also sufferers of major depressive disorders or schizophrenia (Table 1); because these patients often had distorted perception of reality.

In conclusion, the data is important because it is one of few original reports on Asian patients with rodenticide poisoning. Furthermore, the favorable outcome (zero mortality rate) is comparable to the published reports from other international poison centers. Nevertheless, the retrospective nature of the study, the small patient population, and the short follow-up duration are limitations that warrant further investigations to validate the conclusion drawn here.
